# Bioactive Components in Fruit Interact with Gut Microbes

**DOI:** 10.3390/biology12101333

**Published:** 2023-10-13

**Authors:** Yuanyuan Jin, Ling Chen, Yufen Yu, Muhammad Hussain, Hao Zhong

**Affiliations:** 1 College of Food Science and Technology, Zhejiang University of Technology, Hangzhou 310014, China; yuanyuan_j@126.com (Y.J.); 18357183876@163.com (Y.Y.); 2 Sanya Branch of Hainan Food and Drug Inspection Institute, Sanya 572011, China; wanghaotingxue@163.com

**Keywords:** gut microbiota, fruit bioactives, short-chain fatty acids, diseases

## Abstract

**Simple Summary:**

Fruit intake plays a vital role in regulating the gut microbiota; nutrients obtained from fruit sources correlate with microbiota structure and composition. In recent years, the growing evidence indicates that gut microbial diversity is closely associated with human health and disease.

**Abstract:**

Fruits contain many bioactive compounds, including polysaccharides, oligosaccharides, polyphenols, anthocyanins, and flavonoids. All of these bioactives in fruit have potentially beneficial effects on gut microbiota and host health. On the one hand, fruit rich in active ingredients can act as substrates to interact with microorganisms and produce metabolites to regulate the gut microbiota. On the other hand, gut microbes could promote health effects in the host by balancing dysbiosis of gut microbiota. We have extensively analyzed significant information on bioactive components in fruits based on Preferred Reporting Items for Systematic Reviews and Meta-Analysis (PRISMA). Although the deep mechanism of action of bioactive components in fruits on gut microbiota needs further study, these results also provide supportive information on fruits as a source of dietary active ingredients to provide support for the adjunctive role of fruits in disease prevention and treatment.

## 1. Introduction

The gut is considered a “superorganism” comprising up to ten trillion species of microorganisms forming a dynamic and diverse community. The gut is the main microbiota habitat, with the most significant number residing in the distal part [1]. At the phylum level, the microbiota was dominated by five bacterial phyla: Firmicutes, Bacteroidetes, Proteobacteria, Tenericutes, and Actinobacteria [2]. Bacteroidetes and Firmicutes are the two major phyla dominating the gut, followed by Actinobacteria and Verrucomicrobia [3]. Bacteroidetes and Firmicutes phyla are the most diverse and abundant groups of the gastrointestinal microbiota, making up over 80% of the gastrointestinal microbiota of healthy adults [4,5]. Bacteroidetes are closely related to the carbon cycle in the body as many members of them are highly efficient in the degradation of complex carbohydrates [6]. Firmicutes is a dominant phylum in the healthy human digestive tract and can produce butyrate by fermenting non-digestible carbohydrates [7]. *Faecalibacterium*, *Ruminococcus*, *Lachnospira*, *Phascolarctobacterium*, *Roseburia*, and *Dialister* are the major members of phylum Firmicutes. For the Bacteroidetes phylum, Bacteroides, Prevotella, and Alistipes were the most abundant taxa [8,9]. The gut microbiota (GM) is involved in many physiological functions of the host, such as food digestion, nutrient metabolism, immunomodulation, and energy supply to maintain the human host’s health. On the other hand, the host’s health will be impacted by the imbalance of GM in various ways, such as energy absorption, choline, short-chain fatty acids (SCFAs), gut–brain axis, and bile acids (BAs). Once the GM disorder occurs, its structure and function will change and cause the development of some diseases such as inflammation, blood glucose imbalance, and even cancer [7].

GM can affect human health and diseases by interacting with food to produce biologically active substances. Regulating GM through dietary interventions has been popularly suggested as a well-established strategy to maintain health. Fruits are considered as the primary source of active substances. Active ingredients in fruits, such as polysaccharides, dietary fiber (DF), phenolics, flavonoids, and carotenoids could alter and modulate the GM’s variety, quantity, richness, and diversity [10]. Active substances in fruits interact with lactic acid bacteria, which facilitates the diversity of beneficial bacterial groups, changes the ratio of Firmicutes/Bacteroides, and inhibits the growth of harmful bacteria in the gut [11]. Recent studies have shown that some oligosaccharides can alter the composition of the proximal and distal colon microbiota and promote the growth of beneficial bacteria, reducing the amount of *Enterococci* that may have harmful effects, especially *Enterococcus faecalis* and *Enterococcus faecium* [12]. Polyphenols and other metabolites in fruits can increase gut barrier function, reduce oxidative stress, inhibit inflammatory cytokine secretion, and regulate immune function by regulating the GM. Mango, apple, and banana fruit peels are rich in active substances such as total polyphenolics and flavonoids, which can be used as prebiotics and functional ingredients to improve the growth of *Lactobacillus rhamnosus*, *Lactobacillus casei*, and *Bifidobacterium animalis* subsp. *Lactis* [13]. 

**Figure 1:**
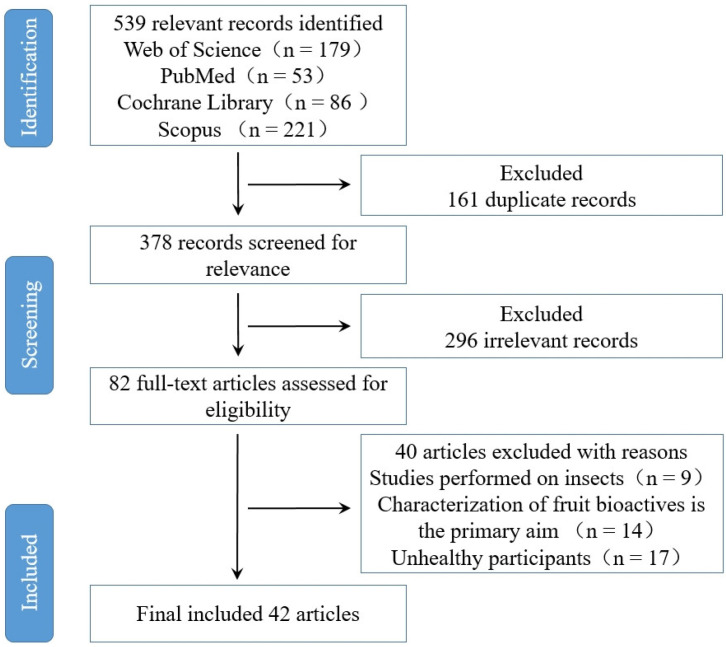
Flowchart detailing the process of identifying and selecting studies.

Based on Preferred Reporting Items for Systematic Reviews and Meta-Analysis (PRISMA) [14], we have extensively compiled, reviewed, and analyzed significant information on bioactive components in fruits from the best published evidence available in PubMed, Scopus (Embase), Web of Science (Web of Knowledge), and Cochrane Library (Figure 1). This review aims to highlight the interactions between fruits and GM, including enriching beneficial flora and inhibiting harmful flora, and to summarize their therapeutic application on gut-microbiota-related diseases and health. We found that the bioavailability and separation efficiency of bioactive components in fruits need to be discussed, and the mechanism of action at the cellular level also needs to be further explored. The purpose of this study was to systematically recover and review the effects of fruit bioactive substances on GM.

## 2. Effects of GM on Human Disease

The GM can be regarded as an independent organ responsible for multiple physiological activities such as host metabolism, neurological development, energy homeostasis, immunological control, vitamin synthesis, and digestion [15]. The GM significantly regulates gut homeostasis, and disruption to the microbiome can cause chronic diseases like gastrointestinal (GI) disorders [16], metabolic disorders [17], and neurodegenerative disease [18]. Table 1 summarizes the effects and mechanisms of GM on human diseases.

### 2.1. GI Disorders

#### 2.1.1. Inflammatory Bowel Disease (IBD)

IBD is a digestive system disorder that affects men, women, and children equally [29]. It is characterized by persistent GI tract inflammation. The two types of IBD, namely Crohn’s disease and Ulcerative colitis, have different inflammation patterns. The GM triggers aberrant host immune responses that lead to IBD [30,31]. The primary environmental factor causing IBD is the GM. A range of 1000~5000 different species may colonize the gut, with Firmicutes, Bacteroidetes, Proteobacteria, and Actinobacteria accounting for 99% of all species [18]. These species alter the ratio of pro- to anti-inflammatory microbes as a critical characteristic of GM dysbiosis, which plays an important role in initiating and perpetuating gut damage [32]. According to human and animal infection model studies, IBD in people is unlikely to be caused or triggered by a single infection. However, it is undeniable that the GM encourages the emergence of IBD [30]. In general, IBD is accompanied by changes in the GM diversity and the production of metabolites and inflammatory cytokines. Reduction in bacterial and fungal colonies and an increase in viral abundance lead to altered GM metabolites such as decreased levels of SCFAs and tryptophan; with increased BAs levels, a transformation of these metabolites leads to the lower intestinal epithelial barrier function and acts on immune cells (especially Treg cells and Th17 cells) to promote the production of inflammatory cytokines [33].

#### 2.1.2. Colorectal Cancer (CRC)

CRC is one of the most common digestive system malignancies [34]. When gut probiotic species (like *Bifidobacteria*, *Lactobacillus*, and *Bacteroides*) decline and the number of pathogenic bacteria (like *Escherichia coli*, *Bacteroides fragilis*, and *Fusobacterium nucleatum*) increases in CRC patients, it indicates the gut microbial dysbiosis. These pathogens may cause tumor necrosis factor while promoting interleukin production, and these signals stimulate immune cells and thus induce the release of cytokines to maintain the balance of the body. Similarly, these can also act on the organism as beneficial bacteria, such as *Faecalibacterium prausnitzii*, which can regulate the specific proliferation of T-cells. Toll-like receptors and NOD-like receptors play a significant mediating process in recognizing microbial signals to activate the immune system [35]. A persistent inflammatory response driven on through the toxic chemicals secreted by the pathogenic bacteria damages gut epithelial cells and contributes to CRC progression [36]. Recent studies indicate that the GM’s toxic metabolites might contribute directly to cancer development or indirectly through immunosuppression or inflammation. On the other hand, imbalances in microbial homeostasis in the gut can also cause changes in metabolite levels that mutate Apc or β-catenin in intestinal stem cells and increase the possibility of normal cell cancer [37].

### 2.2. Metabolic Disorders

#### 2.2.1. Obesity

The incidence of obesity is rising globally, and it is currently considered a global pandemic. Over 1.9 billion persons were overweight in 2020, according to the World Health Organization (WHO). Obesity is associated with GM dysbiosis, which is reflected in the decline in the diversity and richness of the GM in obese persons. The abundance of Phylum Firmicutes significantly increased, while the abundance of *Akkermansia muciniphila*, *Faecalibacterium prausnitzii*, and *Bacteroides* decreased [38]. Some products from GM-fermented indigestible carbohydrates fight obesity by reducing appetite and increasing energy consumption, while others fight obesity by increasing energy consumption and oxidizing lipids.

#### 2.2.2. Diabetes

Diabetes, which includes type 1 diabetes mellitus (T1DM) and type 2 diabetes mellitus (T2DM), is a systemic metabolic illness characterized by excessive blood glucose. A growing number of research suggest that GM is a risk factor in the occurrence and development of diabetes. T1DM is an insulin-dependent type of diabetes [39]. Several research studies show that T1DM patients’ GM significantly differ from healthy individuals. The lower abundance of *Clostridium* and *Prevotella* indicates that the variety of GM in T1DM patients is diminished [40]. T2DM is a non-insulin-independent condition characterized by decreased insulin secretion and resistance [41]. Patients with T2DM have lower levels of *Bifidobacteria* and *Akkermansia* in their guts compared to healthy people, while the abundance of *Dallella* is increased [42].

### 2.3. Neurodegenerative Disease

Evidence shows that GM can influence neurological disease progression and even initiate disease onset when in dysbiosis [43]. Neurodegenerative diseases include multiple sclerosis, amyloid lateral sclerosis, Parkinson’s disease (PD), and Alzheimer’s (AD). Even though each of these diseases has different physiological symptoms, they all share underlying etiologies linked to pathology, the majority of which are connected to normal aging. Interestingly, managing the microbiota may have therapeutic promise for the prevention and treatment of neurodegenerative illnesses because the GM and its downstream effectors broadly intersect many of these pathways [18]. For example, Wang et al. [26] have found that the abundance of pro-inflammatory bacteria such as *Escherichia* and *Enterococcus* in the gut of AD patients was increased, and the inflammatory factors TNF-α and IL-6 were released, while the abundance of beneficial bacteria including *Lactobacillus*, *Bifidobacterium,* and *Ruminococcus* were decreased (Table 1).

## 3. Fruit Bioactives’ Effect on GM and Possible Health Benefits

### 3.1. Fruit Bioactives’ Effect on GM

**Figure 2:**
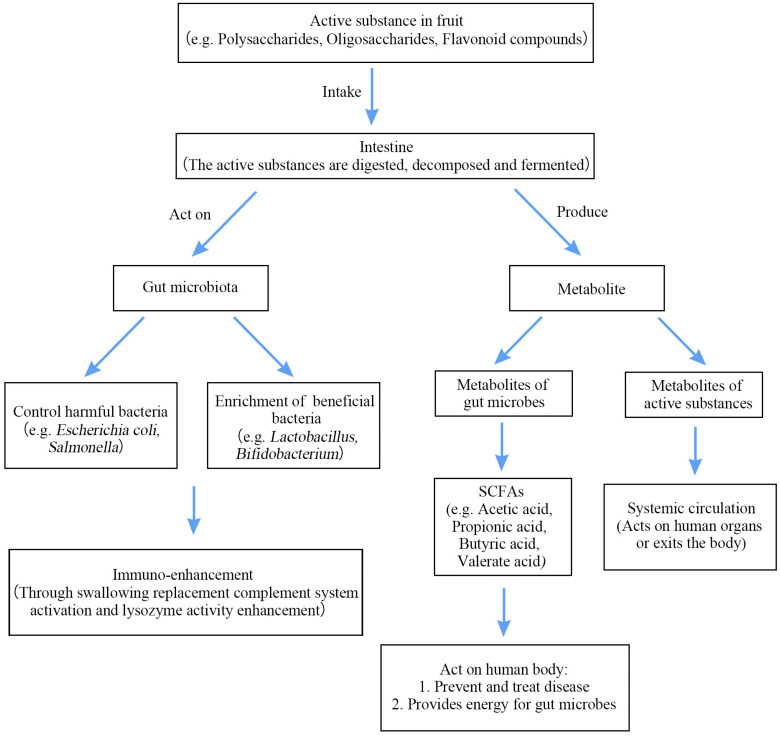
Mechanism of action of bioactive components in fruits.

As shown in Table 2, the addition of fruit bioactive ingredients can significantly regulate the overall structure and composition of GM and further reduce gut dysbiosis. Similarly, the active ingredients in fruits are broken down and metabolized in the gut, and these metabolites enter the human circulation through the bloodstream with great health benefits, as shown in Figure 2.

#### 3.1.1. Polysaccharides

As the most abundant and beneficial dietary components, polysaccharides can modify energy metabolism and regulate host health by influencing GM composition. It has been reported that polysaccharides have antitumor, liver-protecting, anti-inflammatory, antiviral, antioxidant, and anti-aging properties [52,53]. On the one hand, the non-starch polysaccharides can be hydrolyzed by low pH conditions and digestive enzymes in the GI digestion to produce reducing sugars that act as a carbon source for the microbiota. Meanwhile, reducing sugar is also generated from indigestible polysaccharide degradation by the GM. *Aronia melanocarpa* is rich in bioactive components, including triterpenes, flavonoids, phenolic acids, sterols, sugars, and glycosides [52]. The *Aronia melanocarpa* polysaccharide can significantly increase the proportion of beneficial bacteria in the *Bacteroides*, which has a potential role in ameliorating inflammation, retarding the aging process and alleviating cognitive and memory function decline [54]. Tamarind seed polysaccharide was almost completely degraded by fecal microbiota in vitro fermentation culture and further produced SCFAs, resulting in lower pH values. Changes in the gut environment stimulated the increase in relative abundances of beneficial genera, including *Lactobacillus*, *ParaBacteroides*, *Prevotella*, and *Faecalibacterium*. In addition, the polysaccharide treatment reduced gut enteropathogenic genera such as *Escherichia*-*Shigella* and *Dorea*. Thus, tamarind seed polysaccharide has the potential to promote anti-obesity and anti-inflammation as well as to maintain the gut epithelial barrier [54]. Noni (*Morinda citrifolia* L.) fruit polysaccharides can reduce the expansion of pathogenic bacteria, such as *Bacteroides*, *Prevotella*, *Campylobacter*, *Bilophila*, *Escherichia-Shigella*, *ParaBacteroides*, and *Staphylococcus* and increase the relative proliferation of symbiotic bacteria. Overall, Noni fruit polysaccharides significantly reduced the abundance of gut pathogenic bacteria and improved the abundance of beneficial bacteria, and they have useful functions for maintaining the homeostasis of the GM, improving the expression of the gut SCFAs and promoting the repair of the gut mucosal barrier [19]. Homogalacturonan-type pectic polysaccharide from *Ficus pumila* L. fruits induced a significant change in the GM composition, resulting in the Firmicutes/Bacteroidetes abundance ratio, presenting a significant reduction [55]. Pectic polysaccharide regulated metabolite levels and alleviated the disorder of GM, reflected in the increase in myristic levels and pentanolic acids, which were significantly positively correlated with *Akkermansia* and negatively correlated with *Blautia* [56].

#### 3.1.2. Oligosaccharides

Oligosaccharides are considered as potential prebiotics with multiple beneficial effects in regulating GM, enhancing gut barrier strength, and inhibiting gut inflammation. In addition, oligosaccharides offer various benefits, including their production from renewable resources and antimicrobial properties as chemical preservatives. Specifically, their antimicrobial properties can disrupt the cell walls of harmful substances and reduce the adhesion of harmful microorganisms and the action of harmful factors. Moreover, certain natural oligosaccharides have been demonstrated to enhance the growth of beneficial bacteria. In recent years, oligosaccharides applied to the regulation of gut microorganisms are usually extracted from dragon fruit [57], Konjac [57], Lycium [58], and marine algae [59]. Most oligosaccharides cannot be digested in the human GI tract but can be used as a substrate by bacteria in the gut to ferment and convert into SCFAs. However, it has been reported that typical oligosaccharides are primarily fermented by microorganisms in the proximal portion of the colon, producing substantial quantities of SCFAs. Meanwhile, microorganisms in the distal portion of the colon are rich in hydrolytic enzymes, so fermentation in the distal portion of the colon may augment the risk of colorectal cancer. Additionally, oligosaccharides have been shown to change GM’s composition ratio, increasing the proportion of beneficial bacteria such as Lactobacteriaceae, Bacteroidetes, and Firmicutes [60]. Dragon fruit oligosaccharides (DFO) altered the GM composition and increased the number of beneficial bacteria such as *Flavobacterium*, *Flectobacillus*, and *Acidovorax*; on the other hand, they decreased the numbers of *Leptolyngbya* and *Pseudomonas*. Moreover, DFO supplementation significantly increased superoxide dismutase activity, reduced lipid peroxidation, and regulated immune-related gene expression [61]. Galacto-oligosaccharide isolated from mulberry effectively reduced gut dysbiosis, by modulating the abundance and composition of GM, including promoting the growth of Prevotellaceae and *Lactobacillus* while decreasing the abundance of Lachnospiraceae. Furthermore, it was suggested that Galacto-oligosaccharide isolated from mulberry might be a natural anti-diabetic adjuvant drug without side effects, especially GI disturbances [47]. 

#### 3.1.3. DF

DF refers to carbohydrates containing ten or more glycosidic bonds. Based on their solubility, these can be classified as either water-soluble or insoluble DF. Examples of DF include starch, β-glucan, cellulose, and lignin [62]. However, the human gut does not possess the enzymes to break down the majority of ingested DF, while microorganisms in the colon can utilize the undigestible DF for energy via fermentation [63]. Water-soluble DF is more easily degraded and fermented by the GM than insoluble DF. The GI tract benefits from DF by regulating appetite, promoting gut peristalsis, and providing energy to gut epithelial cells. Additionally, it helps to regulate obesity and mitigate the risk of metabolic and cardiovascular diseases in humans. In a double-blind randomized controlled trial investigating the link between DF from fruit and diverticulitis in women, the multivariate risk ratio for diverticulitis was 0.86 among those in the highest quintile of total fiber intake, compared to those in the lowest quintile [64]. Amna et al. [65] conducted a study on the metabolism and GM shift caused by colonic fermentation of DF from ripe and unripe pawpaw. They found that DF from pawpaws triggered SCFA production and a quicker metabolic degradation in ripe pawpaws. It is possible that the higher concentration of water-soluble DF in ripe pawpaw results in the observed effects. Additionally, fermentation in the colon led to an increase in the levels of microorganisms from the *Clostridium* genus, particularly *Clostridium* and *Synechococcus*. Luz et al. [66] created a mango bar rich in DF, which was subjected to in vitro simulation of colonic and digestive fermentation to explore the metabolic effects of DF. The results indicated that SCFAs, in particular butyric acid, and gallic acid levels rose significantly over time, contributing to the bar’s potential to combat oxidative stress and reduce the risk of colon cancer.

#### 3.1.4. Polyphenols

Polyphenols are secondary plant metabolites found mainly in fruits and vegetables. Polyphenols are a large group of chemical components comprising phenolic acids, flavonoids, tannins, lignans, stilbens, and coumarins. Research shows that fruits and vegetables rich in polyphenols have antioxidant properties and free radical scavenging activity, which is beneficial to human health [67]. In addition, polyphenols and their metabolites are critical in regulating GM, enhancing gut barrier function, decreasing oxidative stress, suppressing the release of inflammatory agents, and adjusting the immune system. Recently, people have been interested in increasing polyphenol intake through dietary supplements. Polyphenols are degraded into smaller phenolic acids in the large intestine, affecting the gut’s structure and total number of beneficial species [48]. In a study, Shanthi et al. [68] discovered that polyphenols disparately impact GM’s viability. They inhibit the activity of *Staphylococcus aureus* and *Salmonella typhimurium* while enhancing the adhesion of the probiotic bacterium *L. rhamnosus*. This supports the restoration of GM imbalances caused by stress and other factors and ultimately encourages gut health and overall well-being. Moreover, fermented blackberries can decrease the colonic pH and enhance the production of SCFAs, which are both the cause of changes in the gut ecosystem [48]. Pomegranates are rich in up to 29 kinds of polyphenolic compounds of high content. Research shows that pomegranate fruit pulp polyphenol treatment significantly enriched Bacteroidetes and decreased the abundance of Firmicutes and Proteobacteria. Furthermore, it also shows remarkable increases in the species of *A. muciniphila*, which is consistent with the previous studies of anthocyanin in Açaí (*Euterpe oleracea* Mart.) fruit [49]. Polyphenols present in cranberry fruit with potential antioxidant and anti-inflammatory effects are also shown as therapeutic options for modulating GM [69]. The addition of cranberry increases the relative abundance of the phylum Bacteroidetes, the class Bacteroidia, the order Bacteroidales, and the genera, *Lachnospira* and *Anaerostipes*. Cranberry can also lead to a decline in the relative abundance of order in both Clostridiales and the genus *Oribacterium* [70]. In addition, the active components in cranberry increased the abundance of *Lachnospira* and *Anaerostipes*, contributing to SCFA production [71]. Pomegranate (*Punica granatum* L.) and blueberries are particularly rich sources of dietary polyphenols, which can modulate or be utilized by the GM [72]. Polyphenols are increasingly recognized for their beneficial effects of low-dose dietary habits, as sources of functional food and nutritious food, gaining widespread popularity. Interestingly, some researchers have shown significant interest in fruit polyphenols as antimicrobial agents against foodborne pathogens.

#### 3.1.5. Anthocyanins

Anthocyanins are flavonoids, among phenolic compounds, as a water-soluble pigment that are widely found in plants. Studies indicate that anthocyanins have bioactivities of antimicrobial [73], anti-inflammatory [74], antioxidant [75], immune regulatory [76] and hypoglycemic [77]. However, it should be noted that anthocyanins tend to be inadequately absorbed in the GI tract and hence have low bioavailability. Nevertheless, researchers have hypothesized that anthocyanin metabolites could impact the health implications associated with anthocyanins. Several studies have demonstrated that fruit extracts that are rich in anthocyanins exhibit enhanced bioactivity once they undergo digestion and metabolism in the GI tract. Açaí fruit supplements changed the overall structure and composition of the GM and significantly enriched *A. muciniphila*, thus decreasing the expression of lipogenesis-related genes, resisting lipid accumulation in hepatic adipocytes and restoring liver function [49]. Anthocyanins control body weight by balancing the composition of GM and regulating metabolites. Anthocyanin feeding has been proven to reduce the relative abundance of *Rikenella* and Rikenellaceae. Adjusting the composition and structure of the GM regulates metabolic products, thereby controlling body weight. Research shows that anthocyanin extracted from blueberries and cranberries can modulate the GM by promoting the growth of *Lachnoclostridium*, *Roseburia*, and *Clostridium* innocuum groups in the genus level and further induce the production of SCFAs [78]. Studies show that pomegranate peels are rich in anthocyanins. Pomegranate peel supplements alleviate obesity and related metabolic disorders by changing the composition of the GM, significantly reducing the fasting serum glucose and insulin levels and improving the gene expression profiles involved in glucose and lipid metabolism [79]. Mulberry is rich in amino acids, fatty acids, minerals, and bioactive compounds. Mulberry anthocyanin supplementation can avoid GM dysbiosis to alleviate colitis by reducing the total amount of *Escherichia-Shigella* and increasing the number of *Akkermansia*, Muribaculaceae, and *Allobaculum* [80]. In conclusion, it has been found that anthocyanins can promote the production of SCFAs, accelerate their own degradation, enhance the activity levels of enzymes related to microbes, and promote the proliferation of probiotics. Moreover, it has been demonstrated that physical embedding and molecular modification are successful techniques to enhance the bioactivity and bioavailability of an anthocyanin.

#### 3.1.6. Flavonoids

Total flavonoids can improve the body’s antioxidant capacity, regulate glucolipid metabolism, possess antibacterial properties, and improve the immune system, which results in treating diseases [81]. Flavonoids have low bioavailability and usually act on human health by regulating GM. As reported, 90~95% of polyphenols cannot be absorbed but directly reach the colon, resulting in interactions between GM and polyphenols that benefit the gut cavity and gut mucosa [82]. The flavonoid compounds present in the fruit regulate T-cell differentiation, GM, and gut inflammatory factors, rendering them beneficial in treating inflammatory bowel disease and immune system disorders. In addition, it is worth noting that fruits containing flavonoids can prevent their own decay. This is thought to occur because glycosylated flavonoids inhibit the growth of *Bacillus anthracis*, which plays a role in the fungal infection of fruits and the subsequent hydrolysis via α-glucosidase [83]. Fruits’ intervention did not inhibit the ratio of Firmicutes to Bacteroidetes but reduced the relative abundance of Erysipelotrichaceae, which might be responsible for the anti-obesity effect [50]. Sea buckthorn (*Hippophae rhamnoides* L.) is rich in flavonoids, which has been reported to have the ability to regulate the gut microbiome by decreasing the abundance of Lactobacillaceae and increasing the abundance of Lachnospiraceae at the family level [84]. Total flavonoids from *Chimonanthus nitens* Oliv. leaves can relieve liver inflammation and restore gut homeostasis by regulating GM. As reported, flavonoids from *Chimonanthus nitens* oliv. leaves increased the relative abundance of Firmicutes and Bacteroidetes, while they decreased the relative abundance of Proteobacteria and especially the relative abundance of the family Desulfovibrionaceae, which is an essential group to produce endotoxins [85]. Flavonoids extracted from *Passiflora foetida* fruits promoted the growth of beneficial bacteria, including *Bifidobacterium*, *Enterococcus*, *Lactobacillus,* and *Roseburia*. More importantly, these flavonoids strengthen the gut mucosal barrier and alleviate inflammation by stimulating the production of SCFAs [51]. 

### 3.2. The Efficacy of Fruit-Derived Compounds In Vivo Experiments

**Figure 3:**
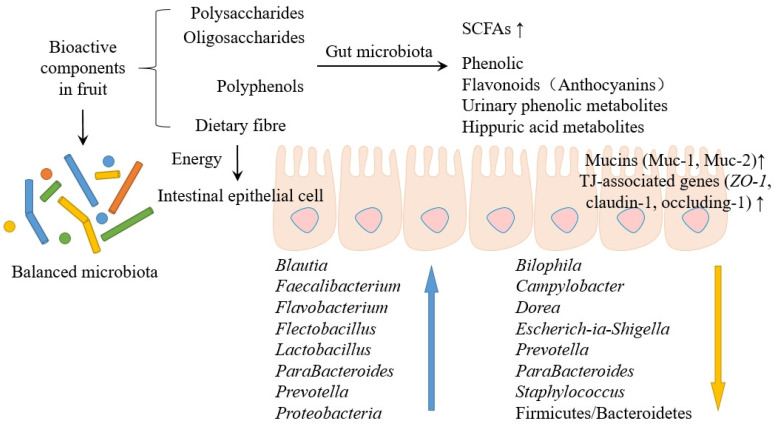
The effects of fruit bioactives on the GM and the cellular environment.

Recent in vivo experiments have confirmed the significant role of fruit bioactive substances in influencing the GM and intestinal cell environment (Figure 3). Li et al. [86] investigated the protective effect of the *Lycium barbarum* polysaccharide (LBP) in preventing ischemia-reperfusion (I/R) injury. They found that LBP inhibited the I/R-injury-induced upregulation of GRK2 expression, and LBP partially restored the I/R-induced mitochondrial fission/fusion imbalance as well as the levels of phosphorylated protein kinase B and phosphorylated endothelial nitric oxide synthase. Liu et al. [12] found that DFO altered the composition of microbiota in the proximal and distal colons, furthermore promoting the growth of beneficial bacteria in the human gut, such as *Blautia*, *ParaBacteroides*, and *Bacteroides*. Results suggest that the number of *Bifidobacteria* and *Lactobacilli* increased significantly due to the utilization of oligosaccharides. Additionally, DFO depleted the numbers of *Enterococci*, especially *E. faecalis* and *E. faecium*, which can lead to infection and have harmful effects on the host. Pansai et al. [87] investigated the effects of DFO on immune stimulation, GM regulation, and the correlation between GM and nutrients in human experiments. The results showed that 4 g/d DFO intake significantly increased IgA levels in healthy adults, and 8 g/d DFO significantly promoted the growth of *Bifidobacterium* spp. and *Faecalibacterium* and reduced harmful bacteria, especially *Escherichia coli*. Mateos-Aparicio et al. [88] used Wistar rats with high-fat diets (HFD) to investigate the potential lipid-lowering effects of apple by-products, mainly portions of soluble DF. It showed potential bifidogenic and butyrogenic effects that are pursued in the search for new prebiotics. It also increased high-density lipoprotein and decreased triglyceridemia and total lipids in the liver, possibly due to BA binding.

Cladis et al. [89] researched that foods rich in polyphenols, such as blueberries and blackberries, can be metabolized by GM to produce phenolic, flavonoids, urinary phenolic metabolites, and hippuric acid metabolites. On the one hand, polyphenols significantly decreased the average Firmicutes to Bacteroides ratio and increased the diversity of Proteobacteria. On the other hand, polyphenolic metabolites can modulate or be utilized by the GM and further increase the diversity and structure of GM. Song et al. [90] investigated the effects of *Averrhoa carambola* L. fruit polyphenols (ACFP) on hyperlipidemic, hepatic steatosis, and hyperglycemia in obese mice induced by HFD. ACFP treatment inhibits the expression of fatty acid synthase and stearoyl-CoA desaturase 1 genes. The expression of fatty acid oxidation-related genes, carnitine palmitoyltransferase 1b and acyl-coenzyme A oxidase 1, was upregulated. In addition, ACFP decreased the expression of phosphoenolpyruvate carboxykinase and increased the expression of insulin receptor substrate 2. Liu et al. [91] demonstrated that *Rosa roxburghii* Tratt. fruit polyphenols (RRTP) change amino acid metabolism, carbohydrate metabolism, and lipid metabolism in mice and regulate SCFA-producing bacteria in the gut. *Blautia*, *Bacteroides*, *Lachnospiraceae_NK4A136*_*group,* and *Roseburia*, as examples, can significantly increase the SCFA content in cecum. These results suggest that RRTP may regulate immune system balance by regulating the balance of metabolites and GM.

Chen et al. [92] studied the antioxidant capacity of anthocyanin Petunidin-3 and 5-*O*-Diglucoside (Pn3G5G) prepared and isolated from *Lycium ruthenicum* Murr. fruit (LRF), and they found that it had a DPPH and ABTS free radical scavenging ability. Oxidative stress damage induced by *N*^ε^-carboxymethyllysine can also be inhibited by clearing ROS and reducing MDA levels. At the same time, the presence of Pn3G5G inhibited the activation of NF-κB and reduced the levels of pro-inflammatory factors. Pn3G5G significantly improved cognitive impairment, neuroinflammation, and neuronal apoptosis in D-galactose-induced aging mice. Villa-Jaimes et al. [93] evaluated *Opuntia robusta* (OR) fruit extract’s hepatoprotective effect on diclofenac-induced acute liver injury. It was found that OR fruit extract decreased the level of oxidative stress by decreasing MDA and GSH; up-regulated the expression of antioxidant-related genes *Nrf2*, *Sod2*, *Hmox1*, *Nqo1*, and *Gclc*; and down-regulated the expression of cell death (Casp3). Zhan et al. [94] analyzed the regulation of 5-Demethylnobiletin (5DN), an important ingredient of citrus extract that is rich in polymethoxyflavones, on antibiotic-associated intestinal disturbances. It was discovered that 5DN could attenuate intestinal barrier injury by increasing TJ expression, including occludin and zonula occluden1. It also modulated the composition of the GM in antibiotic-treated mice by increasing the relative levels of beneficial bacteria, such as *Dubosiella* and *Lactobacillus*.

## 4. Interaction of Bioactive Ingredients with the GM

Bioactive components in fruits interact with the GM to produce a number of metabolites (Table 3), which enter the body’s circulation through the bloodstream and thus exert their health benefits, particularly for immune system and gut health.

### 4.1. Absorption and Metabolism of Bioactive Ingredients in the Intestine

**Figure 4:**
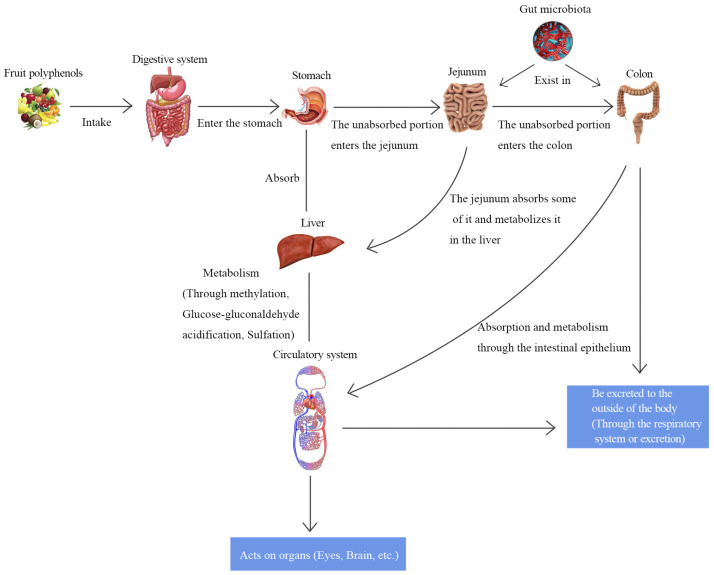
Absorption and metabolism of polyphenols in the intestine.

The interaction between polysaccharides and the human body was found to be facilitated by the GM. The majority of polysaccharides cannot be directly digested and absorbed by the human body due to a lack of polysaccharide hydrolase. GM can convert polysaccharides into lactic acid and SCFAs [111]. *Bacteroides thetaiotaomicron* processes starch using a SUS-like system. This system includes a variety of proteins encoded via genetic clusters of enzymes with different functions (polysaccharide utilization loci) to perform recognition, binding, primary degradation (extracellular), transport (cross-cell and cell intracellular), complete degradation, and signal transmission (intracellular) [112]. Feicalibacteria, on the other hand, is proficient in digesting oligosaccharides and monosaccharides such as fructooligosaccharides, maltose, fructose, glucose, and glucosamine in the absence of extracellular degradation. It introduces two different transport protein types into these sugars for intracellular processing: the ATP-binding cassette and the phosphotransferase system [113]. DF fraction represents the portion of carbohydrates along with lignin not digested by the endogenous enzymes secreted to or present in the small intestine [114]. Nevertheless, because of its physical presence and physiochemical characteristics, DF might affect absorption and digestion in the stomach and small intestine. The intake of DF is directly correlated with the flow of carbs to the large intestine [115]. According to a hierarchical degradation in the large intestine, sugar residues are broken down into oligosaccharides, starch residues, soluble non-starch polysaccharides (NSPs), and finally insoluble NSPs [116]. Flavonoids undergo significant gut metabolism and indicate individual variability. These metabolites comprise smaller phenols with different hydroxylation, glucuronidation, sulphation, and methylation degrees. The common phenolic molecules generated by flavonoid metabolism mainly consist of phenylpropionic, phenylacetic, and benzoic acids with varying degrees of hydroxylation [117]. Similarly, flavonoids and their metabolites in the colon interact with the GM promoting *Bifidobacteria* and *Lactobacilli*, among others, to regulate the GM. The GM, in turn, interacts with the flavonoids to reduce endotoxin production, thereby modulating gut immunity [118]. The biotransformation of polyphenols occurs mainly in the gut, where three main types of chemical reactions occur, mediated by gut microorganisms, namely: (1) carbon–carbon cleavage reactions involving C- and A-rings, (2) dehydroxylations, and (3) hydrogenations [119]. The absorption and metabolism of polyphenols go through two stages. In the first stage of polyphenol hydrolysis, polyphenols undergo hydrolysis, oxidation, and reduction, which leads to the change of polyphenol structure. In the second stage, its biotransformation mainly includes acetylation, methylation, sulfation, and glucuronidation, which leads to the combination of easily available chemical free radicals and phenolic compounds (Figure 4). Take anthocyanins as an example, in the initial phase of anthocyanin hydrolysis, glycosides are formed, primarily due to the splitting of sugar groups. During the subsequent phase, anthocyanins break down into basic phenolic acids within the small intestine, primarily due to the activity of α-L-rhamnosidase and β-D-glucosidase [120]. Victoria-Campos et al. [121] isolated anthocyanins from common fruits, which were separately digested in vitro to study their stability, metabolites, and bioaccessibility, and they found that anthocyanins were catabolized and metabolized to chalcones, glycosides, and ionic-conjugated metabolites under digestive conditions and that their stability mainly depended on the structure of the anthocyanins and the type of glycosylation.

### 4.2. Mechanism of Interaction between Fruits and Gut Microorganisms

#### 4.2.1. Inhibiting the Growth of Harmful Bacteria

Fruit metabolites can directly regulate the internal environment of the intestine and change gut microorganisms’ structure and composition. Anthocyanins can inhibit the growth of pathogenic bacteria, especially *E. coli*, while preventing *E. coli* virulence factors from adhering and invading epithelial cells [122,123]. As reported, Anthocyanins extracted from *Aronia melanocarpa* can destroy the integrity of the *E. coli* cell wall and cell membrane, affecting the cell’s internal environment and leading to cellular metabolic disorders [124]. Pomegranate peel polyphenol is considered a natural, safe, and green antibacterial agent that can inhibit the growth of *E. coli O157: H7* with an inhibition rate up to 95% [125]. Oligosaccharides can protect against *Salmonella* infection by stimulating *Bifidobacteria* and then resisting *Salmonella* colonization by maintaining gut SCFA levels and inhibiting adhesibility [126]. In addition, the degradation product propionic acid can strongly inhibit the growth of *Salmonella*. 

#### 4.2.2. Enrichment of Beneficial Bacteria

The GM is the main factor affecting the microbial barrier. Beneficial bacteria prevent the growth of harmful bacteria and harmful metabolites from posing threats to human health. The enrichment of beneficial bacteria is conducive to maintaining gut microbial homeostasis by resisting the growth of harmful bacteria, such as *Salmonella* and *E. coli*, thus preventing the gut barrier from being damaged. The beneficial ingredients in fruits can enrich beneficial bacteria to regulate GM, such as increasing the abundance and number of *Bacteroides bacteria* that are beneficial to the gut. *Bifidobacteria* is a dominant fraction in the human gastro GM, particularly in infants, that can produce acetate and lactate via sugar fermentation [4]. Generally, pectic-polysaccharides and arabinogalactan can be easily degraded and utilized by *Bacteroides* and *Bifidobacteria* producing a variety of pectin-degrading enzymes, which play an important role in regulating catabolism and gene uptake [127]. Oligosaccharides can promote the growth of *Lactobacillus* and *Bifidobacterium* [128]. Studies show that with the treatment of DFO, *Flavobacterium*, *Flectobacillus,* and *Acidovorax* exhibit relatively high numbers [12]. Polysaccharides from supplementing loquat leaves could significantly change the microbial diversity, structure, and composition of bacterial communities and regulate the proliferation of beneficial microbiota. The polyphenols abundant in plants can further intervene in human disease and health by regulating the GM. Red raspberry, which is rich in polyphenols, can significantly increase abundances of the micronbial families Eggerthellaceae and Clostridiaceae; the genus *Ruminococcus gnavus* was positively correlated with hepatic-IR and *Eubacterium eligens*, and *Bifidobacterium catenulatum* was negatively correlated with cholesterol concentrations [129]. 

#### 4.2.3. Production of Metabolites

In recent years, the primary research metabolites are SCFAs, which mainly consist of acetate, propionic acid, valeric acid, and butyrate, and acetate, propionate, and butyrate are the major SCFAs formed by GM, out of which about 80% chemical properties are different because of the number of carbons [130]. SCFAs are bacteria-derived metabolites, usually produced by indigestible polysaccharides such as resistant starch, fructo-oligosaccharides, simple sugars, and polysaccharides. These polysaccharides are fermented by bacteria in the cecum and colon [78,131]. SCFAs are an important energy source for the epithelial gut cells and provide energy for the growth of microorganisms that directly regulate gut microbial metabolism, playing an important role in maintaining gut homeostasis and health [132]. Firmicutes mainly synthesizing butyrate and *Bacteroides* mainly synthesizing acetate, propionate, *Roseburia*, and *Bifidobacterium* are SCFA-producing genera [129,133]. *A. muciniphila* is a mucin-degrading bacterium and has been identified as the key mucin degrading organism, which produces propionate and acetate and is associated with obesity, diabetes, and many other metabolic diseases [134,135]. *Ruminococcus bromii* could produce butyrate in the colon by fermenting resistant starch [136]. The latest investigations suggest that butyrate prevents hypertension and inflammation [137]. Several recent clinical studies support that SCFAs play a beneficial role in the prevention and treatment of human diseases, such as regulation of cardio-metabolic outcomes [130], improvement of gut barrier integrity [138], regulating cardiac function [139], modulation of glucose and lipid metabolism [140], and mediation of the immune system promoting insulin secretion [141], increasing energy expenditure and reducing fat accumulation [142] and anti-inflammatory response [143]. 

**Figure 5:**
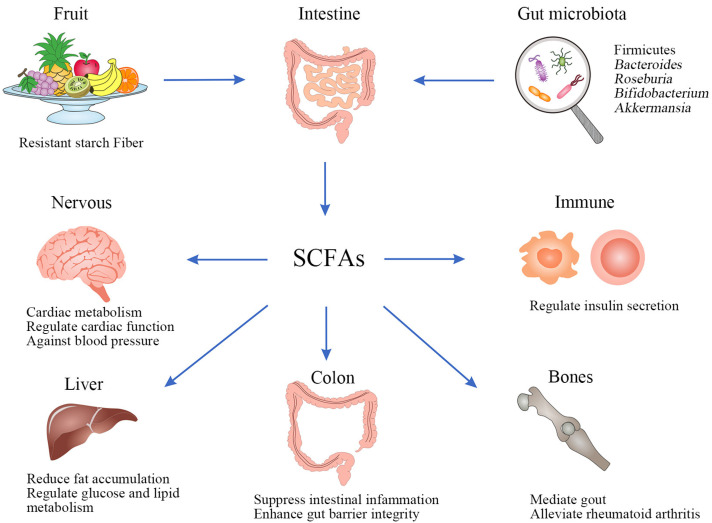
Biosynthesis and beneficial roles in health of SCFAs.

In summary, SCFAs mediate in various pathways and multiple tissues in a concerted action, including immunological, and endocrine influences as well as communication between microbes, gut, and brain (Figure 5).

Dietary SCFAs could alter the core microbiota and enhance mucosal and humoral immune responses by improving specific innate immune parameters and suppressing gut inflammation by modulating their specific receptors [143]. It provides protection against blood pressure by reshaping the composition of the GM and regulating propionate [144]. Based on the gut–lung axis and its association with lung diseases, SCFAs prevent pulmonary disease infections by directly affecting the host immune signaling pathway [145]. The role of SCFAs also extends to peripheral immune function, such as the treatment of arthritis. Acetate has been identified to mediate gout and joint inflammation [146], while the administration of SCFAs could alleviate rheumatoid arthritis [147]. These also prove that the microbiota shapes the host’s ability to respond to extra-gut inflammatory stimuli.

### 4.3. Regulating the Immune System

A host’s immunity may be affected by the changes in the GM, and the type of dietintake has significant effects on the GM. Recent advances in research suggest that the GM may act as a modulator of the efficacy and toxicity of immunopharmaceuticals, particularly through the effects of SCFAs, Bas, and tryptophan metabolites.

Foods rich in fiber and its fermentation give rise to a synergistic effect on the host microbiome and immune system [148]. Prebiotics extracted from fruits, such as fructooligosaccharide, mannanoligosaccharide, inulin, or β-glucan could directly enhance innate immune responses using multiple methods such as phagocytic activation, neutrophil activation, activation of the alternative complement system, and increased lysozyme activity [46]. DFO are utilized as substrates to boost GM growth such as *Flavobacterium*, the dominant genus among Bacteroidetes, which stimulates host growth and improves immunity [61]. Metabolites derived from the GM regulate the development and function of many immune cell types, such as T-cells, B-cells, dendritic cells, and macrophages to improve the host immunity [149]. In a research study, Liu et al. [150] examined the impacts of lingonberry anthocyanins on the gut microbial community and the gut mucosal immune system of mice; they discovered that anthocyanins are capable of enhancing the production of slgA and antimicrobial peptides, which could be due to the increased expression of TGF-β1. Similarly, anthocyanins can alter the composition of gut microorganisms at various levels. They notably reduce the relative abundance of the thick-walled bacteria phylum while increasing the relative abundance of the Anthrobacteria phylum, thereby ensuring the maintenance of gut homeostasis. In a mouse model of IBD induced by dextran sodium sulphate, Jin et al. [19] discovered that Noni fruit polysaccharides could lessen the serum concentrations of LPS, TNF-*α*, and IL-17. Additionally, it inhibits the phosphorylation of JNK, ERK, and NF-κB, whilst promoting the secretion of SCFAs and mucus secretion by cuprocytes in the intestine. It also regulates the disturbed gut microecology in IBD mice, thus enhancing the immunity of IBD mice and alleviating the symptoms of IBD. In a mice model fed with an HFD, the oral administration of cranberry polyphenols and polysaccharides resulted in improved gut homeostasis, which increased the relative abundance of *Ackermannia* spp. while increasing the expression of Toll-like receptor-2 and decreasing the expression of IL-ß1 in mice [151].

## 5. Conclusions

Research over the past decade has shown that GM and its metabolites have a beneficial impact on human health. The fruits contain various bioactive components that influence GM. Some of these components are absorbed by the body directly, while others undergo metabolism to form smaller and more easily absorbed molecules. The fruits also help regulate the GM’s balance, restructure its composition, and rebalance its dynamics. Furthermore, fruits can potentially prevent and treat human diseases, such as neurodegenerative and immune system disorders. 

Numerous studies have objectively reported the direct impact of fruits, their extracts, and by-products on GM. However, it is also vital to comprehend their bioaccessibility and effective isolation from fruits. Previous studies found that the underlying mechanisms by which bioactive substances in fruits act on the GM, such as at the cellular and molecular levels, have not been adequately discussed. In general, understanding the absorption and metabolism of fruit bioactive ingredients in the human body and their effects on the GM is of great significance for enriching disease treatment pathways.

## Figures and Tables

**Table 1:** Influence of GM on human health.

Disease Types	Fruit Bioactives	Microbiota Influence	Others’ Influence	Conclusions	Reference
IBD	Noni (*Morinda citrifolia* L.) Fruit Polysaccharides	Relative abundance of Gram-positive bacteria ↓ Proteus and *Spirospiral* spp. ↓	Acetic acid, propionic acid, and butyric acid ↑ JNK, ERK, and NF-κB phosphorylation ↓ LPS, TNF-α, and IL-17 ↓	Gut microecological balance to inhibit inflammatory signaling pathways.	[19]
IBD	Acacetin, a Natural Dietary Flavonoid	Proteus and *Shigella* spp. ↓	Alleviates body weight loss, diarrhea, colon shortening, inflammatory infiltration, and histological injury TNF-α, IL-1β, IL-6, COX-2, and iONS ↓	Inhibit the inflammatory response and regulate the GM.	[20]
CRC	/	Genus *Klebsiella* ↑ Abundances of *Klebsiella* ↑ Abundance of phylum Proteobacteria ↓	/	There are differences in intestinal microflora between patients with colorectal cancer and normal people after treatment, and the microbial diversity is reduced, which makes them more sensitive.	[21]
Obesity	Total Flavonoids of Quzhou Fructus Aurantii Extract	Genera *Akkermansia*, *Alistipes* ↑ Firmicutes to Bacteroidetes ratio; Genera *Dubosiella*, *Faecalibaculum*, and *Lactobacillus* ↓	Reduced obesity, inflammation, and liver steatosis TC, TG, and OTGG ↓ Phospho-P65, phospho-IKK*α*/*β*, TNF-*α*, and COX-2 ↓	Utilizing prebiotics as dietary supplements to regulate the GM.	[22]
Obesity	Anthocyanin Monomer from *Lycium ruthenicum* Murray. Fruit	Abundances of Bifidobacteriaceae, Helicobacteraceae, and Deferribacteraceae ↑ Abundance of Firmicutes, Lactobacillaceae, Streptococcaceae, Ruminococcaceae, and Erysipelotrichaceae ↓	Control the increase in fat and weight. ALT, AST, TG, and LDL-C ↓ LPS, IL-6, and IL-1β ↓	Anthocyanins can maintain the integrity of intestinal barrier and regulate GM.	[23]
Diagnosed Diabetics	/	*Lactobacillus* ↑ Abundance of *Megasphaera*, *Escherichia*, and *Acidaminococcus* ↑ *Akkermansia*, *Blautia*, and *Ruminococcus* ↓ Abundance of *Sutterella* ↓	LPS and IL-6 ↑	The intestinal flora of diabetic patients at different stages tends to recover to normal people after treatment.	[24]
Diagnosed Diabetics		*Blautia obeum* and *Blautia wexlerae* ↑ *Bacteroides dorei*, *Coprococcus eutactus*, *Akkermansia muciniphila*, and *Bacteroides* spp. ↓	3,8-dihydroxy-urolithin (urolithin A), phenyl-γ-valerolactones, and various phenolic acid concentrations ↓	The change of microbial composition is closely related to diabetes control.	[25]
AD	/	*Escherichia* and *Enterococcus* ↑ *Lactobacillus*, *Bifidobacterium*, and *Ruminococcus* ↓	TNF-α and IL-6 ↑	The abundance of pro-inflammatory bacteria increases, thus releasing inflammatory factors.	[26]
PD	Probiotic Supplement	*Christensenella* spp. and *Marseille-P2437* ↑ *g_Eubacterium_oxidoreducens_group*, *g_Eubacterium_hallii*_*group*, and *s*_*Odoribacter*_sp._*N54.MGS-14* ↓	/	Probiotics treatment can effectively improve the constipation symptoms of PD patients and positively affect the GM.	[27]
Autism Spectrum Disorders	/	Firmicutes, Actinobacteria, *Bacteroidetes*, and Proteobacteria ↑ Faecalibacterium, *Bacteroides*, *Prevotella*_*9*, *Blautia*, and *Subdoligranulum* ↑	/	Gastrointestinal symptoms are positively correlated with autism symptoms, among which constipation is the most common.	[28]

Note: Arrows indicate changes in content or proportion, “↑” indicates increases, “↓” indicates decreases; “/” indicates no mention.

**Table 2:** Effects of GM and body health by bioactives in fruits.

Bioactives	Fruit	Microbiota Influenced	Effect on Health	Reference
Polysaccharide	*Aronia melanocarpa*	*Bacteroides* phylum ↑ Firmicutes ↓	Alleviated inflammation and oxidative stress injury in aging brain tissue	[44]
Polysaccharides	Noni (*Morinda citrifolia* L.)	Enterobacteriales, BetaProteobacteriales, Verrucomicrobiales ↑ Gram-positive bacteria ↓	Promote gut mucosal barrier; improve the expression of gut SCFAs	[19]
Polysaccharide	Pitaya flower buds	Lachnospiraceae, Ruminococcaceae ↑ Muribaculaceae, Lactobacillaceae ↑	Alleviate obesity; prevent gut atrophy	[45]
Oligosaccharides	Dragon fruit	*Flavobacterium*, *Flectobacillus*, *Acidovorax* ↑ *Leptolyngbya*, *Pseudomonas* ↓	Improve immunity; reduce lipid peroxidation and oxidative stress	[46]
Oligosaccharide	Mulberry	Prevotellaceae, *Lactobacillus* ↑ Lachnospiraceae ↓	Decrease the blood glucose	[47]
Polyphenols	Pomegranate fruit	*Bacteroidetes*, *A. muciniphila*, *ParaBacteroides distasonis*, *Bacteroides acidifaciens*, *Mucispirillum schaedleri*, Lachnospiraceae ↑ Firmicutes, Proteobacteria ↓	Alleviated obesity; insulin resistance and hepatic steatosis	[9]
Polyphenol	Blackberry	Firmicutes/Bacteroidetes ↓	Anti-obese	[48]
Anthocyanin	Açaí (*Euterpe oleracea* Mart.) fruit	*A. muciniphila* ↑	Anti-obesity; alleviate liver steatosis; insulin resistance	[49]
Flavonoids	*Rosa davurica* Pall. fruits	Erysipelotrichaceae ↓	Anti-obesity; restoring liver function; reverse gut dysbiosis	[50]
Flavonoids	*Passiflora foetida* fruits	*Bifidobacterium* ↑ *Enterococcus*, *Lactobacillus*, *Roseburia* ↑ *Alistipes*, *Bilophia*, *Enterobacteriaceae* ↓	Anti-inflammatory and GM modulation	[51]

Note: Arrows indicate changes in content or proportion, “↑” indicates increases, “↓” indicates decreases.

**Table 3:** Metabolites of active ingredients in fruits in the gut and associated microorganisms.

Active Ingredients	Category	Representative Compound	Gut Microbes Involved in Metabolism	Metabolite Transformation	Bioavailability	Reference
Polysaccharide	*Malus pumila* polysaccharide	Starch	*Bifidobacteria adolescentis*, *Ruminococcus bromii*	SCFAs (mainly butyrate)	/	[95]
		Pectin	*Bacteroides*, *Lactobacillus*	SCFAs (mainly acetic acid, propionic acid, butyrate)	/	[96,97]
Oligosaccharide	Fructooligosaccharide	/	*Lactobacillus*, *Clostridium*	SCFAs, carbon dioxide, methane, hydrogen	60%	[98,99]
Polyphenol	Ellagitannins	Sanguiin H6	*Coriobacterium*, *Lactobacillus*	Urolithins	/	[100,101]
		Agrimoniin	Eggerthellaceae, *Bacillus gordonae* sp.	Urolithins	/	[102,103]
	Phenolic acid	Gallic acid	Proteobacteria, Firmicutes	Pyrogallic acid	/	[104]
		Chlorogenic acid	*Bifidobacterium*, *Bacillus*	Coumaric acid, benzoic acid, phenylpropionic acid	30%	[105]
Flavonoid	Flavonol	Quercetin	*Streptococcus lutei*, *Lactobacillus acidophilus*, *Clostridium*, *Bacteroides fragilis*, *Clostridium*	Quercetin 3-glucoside, quercetin 7-glucoside	36–53%	[106,107]
		Rutin	*Bacteroides*, *Clostridium*, *Bacillus*	3, 4-dihydroxyphenylacetic acid, rapesin, quercetin-3-*O*-glucoside, phloroglucinol	22%	[108]
	Flavanone	Hesperetin	*Bacteroides*, *Clostridium*, *Bifidobacterium*	7-*O*-glucuronic acid, hesperidin-7-*O*-hesperidin sulfate	/	[109,110]

## Data Availability

Not applicable.
